# Prevalence, Risk Factors, and Molecular Detection of *Campylobacter* in Farmed Cattle of Selected Districts in Bangladesh

**DOI:** 10.3390/pathogens10030313

**Published:** 2021-03-07

**Authors:** Nazmul Hoque, SK Shaheenur Islam, Md. Nasir Uddin, Mohammad Arif, A. K. M. Ziaul Haque, Sucharit Basu Neogi, Md. Mehedi Hossain, Shinji Yamasaki, S. M. Lutful Kabir

**Affiliations:** 1 Department of Microbiology and Hygiene, Bangladesh Agricultural University, Mymensingh 2202, Bangladesh; nazmulh11@gmail.com (N.H.); s_islam73@live.com (S.S.I.); nasirmbsobug@gmail.com (M.N.U.); mdarif38515@bau.edu.bd (M.A.); vetzia.2004.bd@gmail.com (A.K.M.Z.H.); 2 Graduate School of Life and Environmental Sciences, Osaka Prefecture University, Osaka 598-8531, Japan; sbneogi@yahoo.com (S.B.N.); shinji@vet.osakafu-u.ac.jp (S.Y.); 3 Program Specialist (Livestock), Krishi Gobeshona Foundation (KGF), Dhaka 1215, Bangladesh; mehedi.dls@gmail.com

**Keywords:** farmed cattle, *Campylobacter* spp., prevalence, risk factors, PCR, sequencing, Bangladesh

## Abstract

A cross-sectional survey was conducted in selected districts of Bangladesh to estimate prevalence, risk factors, and molecular detection of *Campylobacter* isolates from 540 farmed cattle of 90 herds. As an individual sample, 540 feces, and as a pooled sample, 180 milk samples, 90 feed samples, 90 water samples, 90 manure samples, and 90 animal attendants’ hand-rinse water were collected and tested via culture, biochemical, and molecular assays. A pretested semi-structured questionnaire was used to collect herd-level data on risk factors with the herd owners. The herd-level data on risk factors were analyzed through univariate and multivariate analyses, and a *p*-value <0.05 was considered statistically significant for all analyses. Overall, farm-level prevalence of bovine *Campylobacter* was enumerated to be 53.3% (95% confidence interval [CI]: 42.5–63.9%). The feces sample was found to be a high level of contamination of 30.9% (95% CI: 27–35%) followed by the manure swab (pooled) at 15.6% (95% CI: 8.8–24.7%). *Campylobacter jejuni* was documented as an abundant species (12.6%), followed by *Campylobacter coli* (5.1%), and *Campylobacter fetus* (0.3%). Older farms (>5 years of age), no/minimum cleaning and disinfection practices, along with animal roaming outside of the farm, were documented as significant risk factors for farm-level *Campylobacter* occurrence. Evidence-based control measures need to be taken through stringent biosecurity and hygienic measurement to lessen the load of the *Campylobacter* pathogen in the farm environment and prevent further transmission to animals and humans.

## 1. Introduction

The genus *Campylobacter* includes a divergent group of Gram-negative bacteria responsible for foodborne gastroenteritis all over the world [1,2]. Over 95 million people infected with foodborne diseases were found to be linked with *Campylobacter* globally in 2010 [1], and an estimated 1.5 million people get infections with these pathogens each year in the United States [2,3]. The food animals: for example poultry, cattle, sheep, pigs, and ostriches; pets, including dogs and cats; and environmental sources, are associated with human campylobacteriosis [4]. As enteric and zoonotic pathogens, some *Campylobacter* spp. are well adapted as a commensal in the intestinal tract of various food-producing animals, like ruminants and poultry [5], and act as a reservoir of *Campylobacter* [4].

Amongst the reservoirs of *Campylobacter* species, cattle are considered to be the source of transmission of many human bacterial infections [6]. These bacteria, inhabiting the gastrointestinal tract of many warm-blooded animals, could excrete through the fecal material of about 20% of cattle at a concentration of ∼3 × 10^4^ cfu/g [7]. Farmed cattle infected with *Campylobacter* spp. may shed the bacteria and increase the risk of introduction of infection into animals and humans via the contaminated settings [8]. The most commonly isolated species are *Campylobacter jejuni* and *Campylobacter coli*, as the primary causal agents of bacterial diarrheal disease in high-income countries [9,10]. In addition, Gillespie et al. [11] reported that the majority of human intestinal campylobacteriosis (>90%) cases are related to *C. jejuni* or *C. coli*. However, *C. fetus* is accountable to be a minor contributor of 2.4% of the total reported cases [12]. The prevalence of *C. jejuni* and *C. coli* in dairy cattle varies from 5% to 53%, based on techniques of isolation including relevant determinants such as the age of the animal (young or adult), seasonality, and the type of sample analyzed (feces, intestinal contents, environmental samples, water, manure, etc.).

In *Campylobacter* spp, several copies of rRNA gene loci, namely 5S, 16S, and 23S rRNA, are occupied in different chromosomal locations [13]; of them, the 16S rRNA gene is specific for *Campylobacter* DNA and has been widely used for genus identification [14]. Subsequently, the hippuricase (*hipO*) gene-based polymerase chain reaction (PCR) assay could discriminate *C. jejuni* from the other species [15]. However, cytolethal distending toxin (*cdt*) gene-based multiplex PCR is able to accurately identify *Campylobacter* strains (*C. jejuni, C. coli*, and *C. fetus*) [16,17]. This multiplex PCR assay has been found to be simple, fast, and reliable for the evaluation of *Campylobacter* species [18].

In most cases, human infections are related to the consumption of poultry products, or even direct/indirect contact with animals and birds [19,20,21,22,23,24]. In addition, contact with animals and animal products to spread of organism among animals and even introduction to humans. Therefore, understanding the distribution of *Campylobacter* in farmed animals in Bangladesh was very necessary. This study can facilitate formulating fit-for-purpose and practical control programs for the reservoir animals, and minimizing the burden of enteric infection in humans. Additionally, this is particularly important in low and middle-income countries (LMICs), where the epidemiology of these pathogens is poorly understood [25]. There are many studies that have been conducted in Bangladesh relating to the prevalence and risk factors assessment of the pathogen, including molecular detection of *Campylobacter* in poultry through diversified samplings; nevertheless, detection in cattle yet to be explored. The determinants are normally associated with the *Campylobacter* prevalence both for herd- and animal-level risk factors like herd size, farm with diarrhea, presence of other animals, biosecurity status, farm concentration, season, water supply, type of feed, overcrowding, stress, gender, and weight [26,27,28,29]. Identification of the herd-level risk factors connected with the distribution of *Campylobacter* at the herd and animal levels are required to frame suitable and operative control programs in the low-resource settings.

The temporal variation of *C. jejuni* incidence in cattle has been observed, with the highest shedding in the summer or winter [30,31]. This seasonal pattern may reflect at the highest level in either fecal shedding in the bovine species or exposure to a common contamination source like grazing land [32,33]. The variation of the temporal distribution of *Campylobacter* spp. was documented in dairy cattle. However, research on the prevalence of *Campylobacter* spp. through wide-range sampling in farmed cattle along with molecular detection has not been conducted.

The major changes in livestock rearing, from subsistence herding to the intensive system, has been witnessed since the last decade to minimize the nascent demand of animal origin food [34]. The total livestock population of Bangladesh comprises 24 million cattle, 26 million goats, 3.5 million sheep, 1.5 million buffaloes, and 347 million poultry [35]. Through artificial insemination (AI) with exotic breeds, the productivity of indigenous cattle has been continually increasing since a few decades ago. Therefore, number of crossbred cattle is steadily growing. This practice is leading to the emergence of cattle specific pathogens, like *C. jejuni* lineages, from host-generalized strains. This implies a significant burden of an impotent zoonotic pathogen that can possibly enable human infections [36]. The emergence of such a cattle-adapted *Campylobacter* pathogen through losing the special gene that caused a down-sized genome in the reductive evolution process is widely noticed among bacteria within divergent niches [37]. This is the first conclusive study on *Campylobacter* in the dairy cattle of Bangladesh through wide-range sampling that confirms the prevalence of *Campylobacter* spp. in farmed cattle along with molecular detection of the isolates. The study also evaluated the potential herd-level risk factors associated with the occurrence of *Campylobacter* in the dairy farming practices of Bangladesh.

## 2. Results

### 2.1. Dairy Farm Management Descriptive Statistics

Of the 90 dairy farms, 55.6% (*n* = 50) and 44.4% (*n* = 40) were included from Mymensingh and Dhaka districts, respectively, of which nearly 60% of farms (*n* = 53) were with a herd size of <20 cattle. Of the surveyed farmed animals, the majority (>90%) were Holstein Frisian crossbred cattle, and the rest of them were Sahiwal or Sindhi/Jersey crossbred cattle. Nearly, two-thirds of the farms (62.2%, *n* = 56) were >5 years old and 68.9% (*n* = 62) fed their cattle with prepared feed (noncommercial/ready-made) after purchasing different components. In the meantime, 64.4% (*n* = 58) of farms used antibiotics supplementation in the cattle feed for prophylactic use. About two-thirds of the farmers (64.4%, *n* = 58) had no training on cattle rearing, and 78.9% (*n* = 71) had no knowledge on risk perception on the *Campylobacter* infection if their farmed cattle get access to pasture or freely roam outside. More than half of the dairy farms (57.8%, *n* = 52) were provided animal health care facilities by non-veterinarian professionals (paraprofessional/quack/farmer himself) (Table 1).

### 2.2. Prevalence of Campylobacter spp.

#### 2.2.1. Farm-Level Prevalence

Among the 90 farms, 48 were found to be positive with *Campylobacter* spp. overall via culture and biochemical tests, and finally, molecular assays (PCR). Therefore, a herd-/farm-level prevalence was confirmed as 53.3% (95% CI: 42.5–63.9%), which represented 56% (95% CI: 41.3–70%) and 50% (95% CI: 33.8–66.2%) in the Mymensingh and Dhaka districts, respectively (Table 2). However, herd-level sub-district (Upazila) prevalence ranged from 33.3% to 100%. No significant variation was observed in the herd- level *Campylobacter* spp. distribution among districts and sub-districts (Upazila) with a *p* value of >0.05 (Table 2).

#### 2.2.2. Sample-Level Prevalence

The different samples (*N* = 1080) that comprised the individual sample are as follows: the overall sample of feces (*n* = 540) and pooled samples of milk (*n* = 180), feed (*n* = 90), water (*n* = 90), manure swab (*n* = 90) and hand-rinse water of the animal attendants (*n* = 90) were collected from 90 dairy farms. Of the 1080 samples, 207 were found to be provisionally positive via a culture-based method, and finally, 194 samples were confirmed as *Campylobacter* spp. by biochemical and molecular tests (Supplementary Figure S2); therefore, an overall sample-level prevalence of 18% (194/1080) was confirmed. The highest prevalence was observed in feces (30.9%) (as an individual sample), followed by manure and hand-rinse water as 15.6% and 11.1%, respectively (as pooled samples). The feed and water samples were found to be a non-contamination status in this study. The distribution of *Campylobacter* spp., in different categories of the sample was found to be associated with the *Campylobacter* positive status (*p* = 0.000). In this study, the highest prevalence was observed in cows (41.1%), followed by calves (28.3%), and heifers (23.3%); this animal-level distribution was found to be statistically significant (*p* = 0.0008) through feces sample evaluation. The highest occurrence of *Campylobacter* (34.6%) was captured in the monsoon season, followed by winter (31%), and pre-monsoon season (29%). However, these temporal variations were found to be non-significant in this study (Table 2 and Table S1).

### 2.3. Molecular Detection of Campylobacter spp.

All *Campylobacter* isolates (194) presented a specific amplification of 1530 bp fragment size via a genus-specific (16S rRNA gene) polymerase chain reaction (PCR) (Figure S1-a), and further molecular detection for *C. jejuni* was accomplished by a *hipO* gene-based PCR that generated an amplicon size of 735 bp (Figure S1-b).

Finally, *cdtA* gene-based multiplex PCR was carried out for the detection of *C. jejuni*, *C. coli*, and *C. fetus.* In this PCR assay, C. *jejuni*, *C. coli,* and *C. fetus* generated 631 bp, 329 bp, and 489 bp amplicon sizes, respectively, as a confirmatory test for species identification (Figure S1-c). Additionally, among the PCR-positive isolates (194), *C. jejuni (n* = 17), *C. coli* (*n =* 1), and *C. fetus (n =* 3) were used for partial sequencing of the 16S ribosomal RNA (16S rRNA) gene, and the interpretation of the sequencing data validated the PCR results. Moreover, the sequences that produced significant alignments were used in the Basic Local Alignment Search Tool (BLAST) analysis, and our study isolates represented an identity of 99.55–100%, 99.79–100% and 96.87–100% for *C. jejuni, C. coli*, and *C. fetus,* respectively.

The data of the partial sequence of the 16S rRNA genes were submitted in the GenBank and the accession numbers were obtained accordingly as *C. jejuni*: H3/ MT782639, H1/MT783398, B1/MT784200, B2/MT784199, B4/MT783401, B3/MT783402, D8/MT783426, D12/MT783690, D2/MT784146, D1/MT784147, D4/MT784163, D7/MT784192, D6/MT784193, D5/MT784195, D13/MT784196, D10/MT784197, D9/MT784198; *C. coli*: H2/MT774557; and *C. fetus*: B5/MT783400, D3/ MT783688, D11/MT783689.

**Figure 1:**
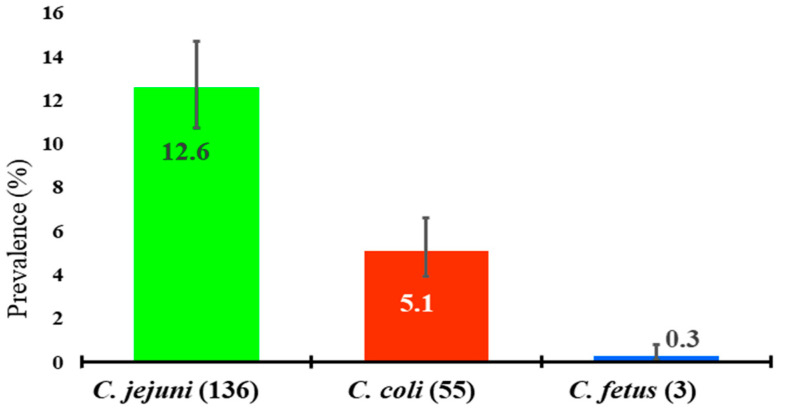
Distribution of isolates (*n* = 194) of *Campylobacter* spp. with 95% confidence interval (CI) that represented the prevalence of *C. jejuni, C. coli*, and *C. fetus* as 12.6%, 5.1%, and 0.3%, respectively, in selected dairy farms confirmed through 16S rRNA, hippuricase (*hipO*), and *cdtA* gene-based polymerase chain reaction (PCR) assays.

In this study, among the 194 isolates, 70.1% (*n* = 136), 28.4% (*n* = 55), and 1.5% (*n* = 3) were confirmed as *C. jejuni, C. coli*, and *C. fetus*, respectively. The distribution of *C. jejuni, C. coli*, and *C. fetus* were captured as 12.6% (95% CI: 10.7–14.7%), 5.1% (95% CI: 3.9–6.6%), and 0.3% (95% CI: 0.1–0.8%), respectively, in the different samples (Figure 1).

### 2.4. Evaluation of Risk Factors

#### 2.4.1. Univariate Analysis

A total of 24 determinants (Table S3) relating to farm management, environmental factors, and biosecurity and hygienic practices were used in univariate analysis. In univariate analysis, farm management and environmental determinants, namely, the age of the farm, the farmers’ training, knowledge on risk perception of animals roaming outside of the farm, animal health service provider, and the floor condition of cattle shed were significantly associated with the herd-level *Campylobacter* status (Table 1). Additionally, biosecurity-related determinants like cleaning and disinfection practices (C&D), other animals’ access (birds/goats/sheep/wild animals) to the farm, and animals roaming outside of the farm were confirmed as significant risk factors for *Campylobacter* status in invariable analysis (Table 3).

#### 2.4.2. Multivariate Analysis

Among the risk factors, nine were included in the multivariable logistic regression analysis, as these were found to be statistically significant in univariate analysis. The risk factors for herd-level *Campylobacter* status were identified in the final multivariable logistic regression model. The most important risk factors associated with *Campylobacter* positive status were identified as older farms (more than 5 years), no/minimum cleaning and disinfection practices, and animals roaming outside (Table 4). The odds of *Campylobacter* positive status were 10.6 times (95% CI = 1.9–59.8, *p* = 0.0007) higher in a cattle farm with an age of > 5 years compare to a cattle farm of the age of 1–5 years. The cattle farm had no/minimum cleaning and disinfection practices had 12.4 times (95% CI: 2.1–71.6, *p* = 0.0048) higher risk to be *Campylobacter* positive status. The farms with roaming animals were 44.0 times (95% CI: 3.6–537.0, *p* = 0.0048) more likely to be positive with *Campylobacter* compared to farms with no roaming animals (Table 4).

## 3. Discussion

In this study, we evaluated herd-level *Campylobacter* status in the light of prevalence and the molecular detection of *Campylobacter* isolates, and assessed the risk factors for herd-level occurrence in the farmed animal in two cattle-dominant districts in Bangladesh. We evaluated the overall herd-/farm- and sample-level prevalence of *Campylobacter* spp. as 53.3% (48/90) and 18% (194/1080), respectively. However, several studies in different geographical locations confirmed high herd-level prevalence of *C. jejuni* and/or *C. coli* as 78.8% in beef cattle and 86.6% in dairy cattle in northern Spain [38]. Moreover, a low herd-level prevalence (33%) was reported in Austria [28].

A high level of prevalence of *Campylobacter* spp. in feces (30.9%, 167/540) among all categories of the tested sample was documented. This finding is consistent with an earlier study conducted in Bangladesh, as the prevalence of *Campylobacter* spp. was documented as 26.7% and 20% in feces and milk samples, respectively, that were collected from cattle (*n* = 40) [39]. This finding is consistent with a study conducted in Odisha, India, in which 25.33% of fecal samples collected from farmed animals (cow, sheep, and goats) showed the prevalence of *Campylobacter* spp. [40]. A high-level prevalence of *Campylobacter* spp. in feces was reported in different studies as 87%, 69.1%, 78%, 66.7%, and 78.5% in Canada, France, Sweden, Basque Country, and Lithuania, respectively [26,41,42,43,44]. However, lower levels of prevalence were confirmed in Asian countries, as 14% in dairy cows in Thailand [45] and 1.6% in buffaloes in Lao People’s Democratic Republic [46]. Moreover, a low-level of prevalence of 14.9% in feces was recorded in Austria [28]. However, because of limited published reports on *Campylobacter* distribution in farmed cattle, this evaluation was not fit to compare in Bangladesh.

We confirmed the prevalence of *C. jejuni*, *C. coli*, and *C. fetus* for 12.6% (136/1080), 5.1% (55/1080), and 0.3% (3/1080) of samples, respectively. However, higher prevalence of *C. jejuni* was detected as 25.6% from a dairy farm sample in Korea [47] and 69% from beef cattle feces in Canada [26]. Among the isolates, we verified that 70% (136/194), 28% (55/194), and 2% (3/194) were from *C. jejuni, C. coli,* and *C. fetus,* respectively. This result is consistent with the findings of a study conducted in Sweden [42], as 61% of isolates were *C. jejuni,* including a negligible proportion of isolates (0.7%) that were confirmed as *C. coli*, which disagreed with our study findings. Moreover, the present study confirmed 11.1% (10/90) of the hand-rinse water of the animal attendants was found to be contaminated with *Campylobacter* spp. Therefore, our study findings present the wide distribution of *Campylobacter* spp. in farm settings that might be responsible for the transmission among animals, from animals to humans, or even prevalence that is successfully maintained within the farms’ environments.

The study documented that 15.6% (14/90) of manure (pooled) samples were positive with *Campylobacter* spp., and the proportion of *C. jejuni* was found to be higher (70%, 136/194) than the other isolates. *C. jejuni* survives at lower temperatures, rather than at higher temperatures, which signifies the risk of contamination to other foodstuffs [48]. Moreover, studies have confirmed that *Campylobacter* are able to survive in a very harsh environment, like a hot and humid environment, or even in a manure compost pit [49,50]. These factors would facilitate to the subsistence of *Campylobacter* in the farm environment for a longer period of time.

In this study, 1.7% (3/180) milk samples were found to be contaminated with *Campylobacter* spp. A few studies confirmed the high level of milk filter samples that were positive with *Campylobacter* as 14% and 13% in Sweden [42,51]. Studies have established the risk of the introduction of *Campylobacter* spp. through the consumption of raw milk in different geographic locations [52,53,54,55].

The farm-level prevalence of *Campylobacter* spp. did not differ among the districts (*p* = 0.57) and sub-districts (*p* = 0.64). However, the sample-level prevalence of *Campylobacter* spp. was found to be significant (*p* = 0.000). The distribution of *Campylobacter* for different age groups (cows, heifers, and calves) of cattle was assessed significant (*p* = 0.0008) (Table 3). In this survey, approximately one-third of cattle farms (30.9%) were found to be positive with *Campylobacter* by feces sample evaluation. This could be a source of infection for humans through direct contact [56,57] or environmental contamination. In this regard, control measures need to be adopted through mandatory cleaning and disinfection practices. The odds of becoming *Campylobacter* positive were 12.4 times higher with farms that had poor to no cleaning and disinfection practices. This finding is supported by another study as low-to-moderate cleaning and disinfection practices had 9.24 times more likelihood to be *Campylobacter* positive [47]. Appropriate hygienic measurements in cattle farm and milking points, and cleanliness practices of dairy cattle sheds, can reduce the growth and subsequent transmission of *Campylobacter* spp. [43,58,59].

Farmed animals allowed to freely roam outside and pasture graze increase the likelihood of exposure to multiple sources of contamination [43]. The farms with freely roaming animals have more probability (AOR = 44.0, 95% CI: 3.6–537.1, *p* = 0.003) to be positive with *Campylobacter* spp. This finding is supported by other researchers who showed that grazing cattle have a higher likelihood to be positive with *Campylobacter* infection [60,61]. The present study confirmed that older farms (>5 years) are more likely to be *Campylobacter* positive. This might be potential to successful maintenance of organisms for a longer period of time if cleaning and disinfection practices are not performed properly in the cattle farm. The fact that older broiler farms (>15 years) have a higher likelihood of *Campylobacter* occurrence was established in six European countries [62]. However, due to lack of reference data in cattle that makes inconclusive our study findings to compare. In this study, the occurrence of *Campylobacter* in dairy farms was found to be marginally higher in the monsoon season compared to the winter and the pre-monsoon seasons, but this was found to be non-significant. The temperature variation in different seasons is minimum, as Bangladesh has a hot, humid, warm, tropical climate with mild winters [63], which might lead to the non-significance of temporal impact on the variation of *Campylobacter* occurrence at the farm level.

In this study, some potential variables were shown to be non-significant with the farm-level *Campylobacter* status in the multivariable logistic regression model, i.e., the animal shed, farmers’ training, knowledge, the animal health care provider, the floor condition, and access of other animals (poultry/goats/sheep/wild animals). Farmers’ knowledge of risk perception on how *Campylobacter* is released, maintained, and transmitted is needed as compliance with the biosecurity practices [64]. The training of animal attendants/farmers related to biosecurity and hygienic measurement has been documented to reduce *Campylobacter* exposure and further maintenance at the farm level [65,66]. Animal health care services (vaccination, medication) provided through non-veterinarians (paraprofessional/quack/farmer) were found to be risky practices that were likely to be associated with *Campylobacter* infection [67]. Access by other animals (poultry/goats/sheep/wild animals) in the farm premises facilitates to the introduction of *Campylobacter* was also investigated [28].

The study confirmed herd-level *Campylobacter* spp. status on the basis of feces sample evaluation. However, several pooled samples, like swabs from the manure pit, water, feed, and the hand-rinse water from cattle attendants, were collected from the same herd during sampling to confirm the feces test results. This also signifies a diverse distribution of *Campylobacter* species within the same group (herd) of cattle. This study depicts the levels of *Campylobacter* distribution in dairy farming practices that included the herd- and animal-level occurrence with potential risk factors. This signifies that source tracing of *Campylobacter* spp. in food animals is necessary. There is an urgent need for surveillance of *Campylobacter* in the farm environment, as they change with time [68], and host-generalized strains may develop through intensive cattle farming [36] as the farmed cattle population gradually increases in Bangladesh. Therefore, appropriate preferences to lessen the burden of *Campylobacter* through good farming practices that include biosecurity and hygienic practices and better management of cattle excreta are needed.

This study has a few limitations, as the culture evaluation and subsequent molecular detection were done using a single colony from each sample of the subculture. This signifies the samples with more than one species of *Campylobacter* could not be identified under this study, and the blood agar-negative samples did not culture in the broth-based media, which may have reduced the sensitivity of our primary evaluation of *Campylobacter*. The study mostly used herd-level determinants for identifying the *Campylobacter* positive status, which created tailbacks to explore the risk factors conclusively. Therefore, a future study including all levels of risk factors for confirming the determinants of *Campylobacter* occurrence in dairy cattle, and corresponding sampling in humans, is warranted.

## 4. Materials and Methods

### 4.1. Study Location, Design, and Survey Farms

**Figure 2:**
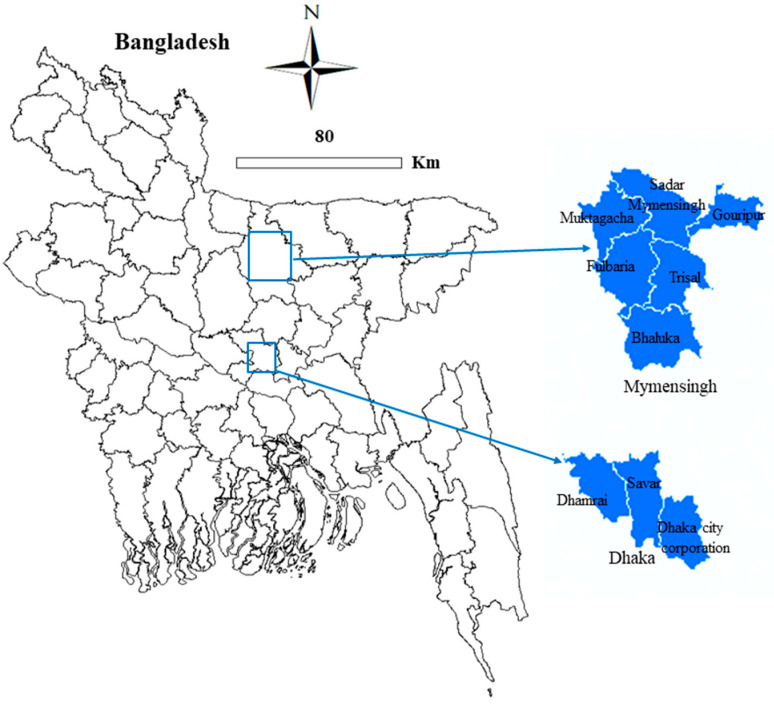
Location of study districts (Mymensingh and Dhaka). A total of six sub-districts (Mymensingh Sadar, Muktagacha, Gouripur, Fulbaria, Trisal, and Bhaluka) of the Mymensingh district and two sub-districts (Dhamarai and Savar) and the Dhaka City Corporation (DCC) area of the Dhaka district were included in this study.

A cross-sectional survey was conducted in commercial crossbred (Holstein Frisian & Shahiwal Crossbred) farmed cattle of Dhaka and Mymensingh districts from April 2018 to May 2020 (Figure 2). The study sites were selected on the basis of animal distribution, as these districts are considered to be promising cattle-rearing zones of Bangladesh. There are 226,000 and 923,000 heads of cattle in Dhaka and Mymensingh districts, respectively [69], of which 15% are crossbred cattle [70]. Six sub-districts (Mymensingh Sadar, Muktagacha, Gouripur, Fulbaria, Trisal, and Bhaluka) from the Mymensingh district, and two sub-districts (Savar and Dhamrai) and the Dhaka City Corporation (DCC) area from the Dhaka district were included in this survey. There are around 300 registered dairy farms in the two districts, of which 90 farms from the districts (Mymensingh: 50; Dhaka: 40) were randomly surveyed after consultation with local (sub-district/municipality) livestock offices.

### 4.2. Face-to-Face Data Collection in Field Survey

A pretested semi-structured questionnaire (Table S3) was designed and used for data collection from farmers/farm attendants during sampling from the farm. The questionnaire had 24 questions in two broad areas: (i) variables related to farm management and environment factors (15 questions); (ii) factors related to farm biosecurity and hygienic measurements (9 questions). The questionnaire was translated into local dialects and used in a face-to-face interview session so that the respondent could easily understand its content. However, some data were collected through the field observation/ transect walk method. The responses from the respondents were coded and recorded in Excel spreadsheets for further analysis (Table S2).

### 4.3. Sample Size and Sampling Procedure

#### 4.3.1. Sample Size Calculation

Multi-stage random sampling was done in this study through an initial selection of farms, and subsequently, sampling was done in different categories (calves, heifers, and cow) of animals. The sample size was calculated using the formula given below Equation (1) [71].

(1) $$n=Z^{2}p\left(1-p\right)/d^{2}\left(1\right)$$

where *n* denotes the required sample size, *Z*^2^ is the *Z*-score at a 95% confidence interval or 1.96, *p* is the expected prevalence of *Campylobacter* at the animal level (27% = 0.27) [39], and *d* is desired absolute precession (4% = 0.04); thus, a sample size of 474 was obtained. However, we included 540 animals for sampling from the two study districts.

#### 4.3.2. Sample Collection from Animals

A total of six individual animal fecal samples were collected from each farm that consisted of two from cows, two from heifers, and two from calves. Additionally, as pooled samples, milk (*n* = 2), feed (*n* = 1), water (*n* = 1), manure swabs (*n* = 1), and the hand-rinse water of farmers/farm attendants (*n* = 1), were collected from each farm. To avoid sampling bias in each pool sample category of feed, water, manure, and hand-rinse water, three sub-samples were randomly collected and pooled together as the “pooled sample”. In sum, 1080 samples were collected from 90 dairy farms that comprised 540 feces (as an individual sample) samples, and 180 milk, 90 water, 90 feed, and 90 animal attendants’ hand-rinse water samples (as pooled sample) that were collected from the two districts (Table S1). Herd-level prevalence was confirmed on the basis of the feces sample evaluation status, and the status of the pooled samples was evaluated to verify the herd-level occurrence.

Aseptic precautions were maintained during the collection of the samples. The amount of sample varied according to the sample type as a 1–5 mL or g swab material for the feces and manure swab samples, 100 g for the feed sample, and 100 mL each for the water, hand-rinse water, and milk samples. The swab samples were preserved and transported in Cary-Blair transport media. Samples were retained in a plastic container (100 mL falcon tubes and plastic polybags) immediately after collection with a given unique identification number, and transferred to the laboratory of the Department of Microbiology and Hygiene, Bangladesh Agricultural University (BAU), Mymensingh, while maintaining a cool chain at 4–6 °C.

### 4.4. Laboratory Evaluation

#### 4.4.1. Culture and Biochemical Tests

All samples were analyzed individually by filtration method using the cellulose filter with a porosity of 0.45 µm (Biotech, Göttingen, Germany). This size is effective to hold 90% of cells [72] with high-flow rates and enable the best colony growth. The culture of *Campylobacter* was carried out in selective media with the procedure described earlier [72] with little modification. Briefly, 100 µL of each collected sample was spread on the filters that were placed on the surface of blood agar base no. 2 (HiMedia, Mumbai, India) (supplemented with 5% sheep blood), with Skirrow supplement (for both *C. jejuni* and *C. coli*) (HiMedia) and/or with growth supplement (for *C. fetus*) (HiMedia) and allowed to stand for 30 min at room temperature. After 30 min we removed the filter from the Skirrow and/or growth supplement blood agar and then incubated the plates at 37 °C for 48 h in microaerophilic conditions (5% O_2_, 10% CO_2_, and 85% N_2_) using AnaeroPouch^®^-MicroAero (Mitsubishi Gas Chemical Co., Inc., Tokyo, Japan). After 48 h, the incubated media were then examined for the growth of bacteria. Grey, flat, and irregularly spreading colonies were observed on the surface of the media. The colonies were then subjected to Gram’s Method of staining and observed under a microscope for Gram-negative curves. The selected colonies from the selective agar media were then subcultured onto the supplemented blood agar base no. 2 to obtain a single and pure colony. Differentiation of isolated *Campylobacter* spp. based on growth characteristics including biochemical tests, such as the catalase test, oxidase test, and hippurate hydrolysis test, were performed according to the standard procedures described earlier [73,74,75].

#### 4.4.2. Molecular Detection through PCR

Culture-positive isolates were further confirmed as *Campylobacter* spp. by biochemical tests and PCR assays. The DNA was extracted from the pure culture of *Campylobacter* spp. by boiling method [76]. The genus of *Campylobacter* was verified through the amplification of the 16S rRNA gene using oligonucleotide primers, as per the procedure described in Table 5 [76].

In this study, the identification of *C. jejuni* was accomplished by two molecular assays. After the primary confirmation of *Campylobacter* spp., hippuricase (*hipO)* gene-based PCR was done using all the isolates to discriminate *C. jejuni* as per the method defined in Table 5 [18]. Secondly, *cdtA* gene-based multiplex PCR assay was performed for *Campylobacter* identification by species (i.e., *C. jejuni, C. coli*, and *C. fetus*) using all isolates (*n* = 194) as per the method described in Table 5 [77]. In PCR assays, positive controls and DNA templates of *C. jejuni* ATCC 33560, *C. coli* ATCC 33559, and *C. fetus* ATCC 27374 strains were used. *Escherichia coli* ATCC 25922 was used as a negative control (Supplementary Figure S1). Information of all primers and corresponding PCR amplicon sizes are presented in Table 5. PCR products were visualized at gel electrophoresis (1.5% agarose, Invitrogen, Carlsbad, CA, USA), and after staining with ethidium bromide (0.5 μg mL^−1^) and destaining with distilled water for 10 min, further gel images were taken using a UV transilluminator (Biometra, Göttingen, Germany).

#### 4.4.3. Sequencing of 16S rRNA Gene

The primers used for the sequencing of the 16S rRNA gene of *Campylobacter* species (*n* = 21) are presented in Table 5. After amplification of the 16S rRNA gene, the PCR product was purified using a Wizard^®^ SV Gel and PCR Clean-Up System according to the manufacturer’s instructions (Promega, Madison, WI, USA). The purified PCR products were sequenced through standard Sanger’s sequencing method with the BigDye terminator v3.1 sequencing kit and a 3730xl automated sequencer (Applied Biosystems, Foster City, CA, USA). Homology searches were accomplished against highly similar sequences (megablast) in the GenBank database using the BLAST analysis tools, which are available from the National Center for Biotechnology Information (NCBI) website (https://www.ncbi.nlm.nih.gov/) (accessed on 10 December 2020). Finally, the sequences were deposited to the GenBank, and accession numbers were obtained against each sequence.

### 4.5. Statistical Evaluation

In this study, the unit of analysis was a herd or farm. A herd/farm was considered to be positive if the individual feces sample collected from each animal tested positive in both tentative (culture-based and biochemical tests) and confirmatory molecular assays (PCR). Therefore, the dependent variable of this study was dichotomous data, either *Campylobacter* positive or negative. Several continuous variables were converted into categorical variables (age of farm, farm size, stocking density) to accomplish the analysis.

Data from field surveys and laboratory evaluations were recorded in Microsoft Excel 2010 (MS Excel) spreadsheets and data were cleaned, coded, and checked for consistency. The data were further exported into the Epi Info 7 program [79] for statistical analysis. The odds ratio (OR) was calculated through univariate logistic regression model for estimating the relationship on *Campylobacter* positive status, and a *p*-value of <0.05 was considered as statistically significance. The significant variables were further utilized in the multivariable logistic regression model. Descriptive analysis was done, the outputs were presented in frequencies and proportion, and 95% binomial confidence intervals (CI) were confirmed using the excel data analysis tool pack for estimating the prevalence values of *Campylobacter* spp. in various samples and at the farm level. Categorical response variables were presented as proportions, and their associations determined by Pearson’s Chi-square tests.

## 5. Conclusions

Cattle have been recognized as reservoirs of *Campylobacter* that facilitate environmental contamination through feces. This finding suggests the need for appropriate control measures to promote good animal husbandry practices, including stringent biosecurity and hygienic measurements. The importance of participatory training and good farm practices for cattle farmers and attendants highlights the environmental, animal, and human “One Health” approach to mitigate the prevalence of *Campylobacter* in the farm environment and prevent further transmission to animals and humans.

## Figures and Tables

**Table 1:** Univariate analysis of farm management and environmental factors associated with the herd-level *Campylobacter* prevalence in cattle farms of Dhaka and Mymensingh districts.

Variables	Category	Number of Positive Farms (%)	Odds Ratio	95% Confidence Interval (CI)	*p* Value
Farm location (District)	Mymensingh (*n* = 50)	28 (56)	1		0.57
	Dhaka (*n* = 40)	20 (50)	0.8	0.3–1.8	
Age of the farm	Up to five years (*n* = 34)	13 (38.2)	1		0.03
	>5 years (*n* = 56)	35 (62.5)	2.7	1.1–6.5	
Animal shed	Newly constructed within a year (*n* = 24)	8 (33.3)	1		0.021
	Old (more than one year) (*n* = 66)	40 (60.6)	3.1	1.1–8.2	
Farm (herd) size	Up to 20 cattle (*n* = 53)	28 (52.8)	1		0.90
	>20 cattle (*n* = 37)	20 (54.0)	1.1	0.4–2.4	
Stocking density	More than 50 sq. ft./animal (*n* = 49)	18 (36.7)	1		0.63
	Less than 50 sq. ft./animal (*n* = 41)	23 (56.1)	1.2	0.5–2.8	
Milking type	Machine milking (*n* = 5)	2 (40)	1		0.53
	Hand milking (*n* = 85)	46 (54.1)	1.7	0.3–11.1	
Feed used	Readymade feed (*n* = 28)	15 (53.6)	1		0.975
	Prepared by farmer (*n* = 62)	33 (53.2)	0.9	0.4–2.4	
Farmers’ training	Yes (*n* = 32)	11 (34.4)	1		0.0007
	No (*n* = 58)	37 (63.8)	3.4	1.4–8.3	
Knowledge on risk perception of cattle access outside or freely roaming	Yes (*n* = 19)	5 (26.3)	1		
	No (*n* = 71)	43 (60.6)	4.3	1.4–13.3	0.007
Cattle handler type	Family member (*n* = 28)	16 (57.1)	1	0.3–1.9	0.62
	Employee (*n* = 62)	32 (51.6)	0.8		
Prophylactic use of antibiotics	Yes (*n* = 58)	30 (51.7)	1	0.50–2.8	0.68
	No (*n* = 32)	18 (56.2)	1.2		
Animal health care provider	Registered veterinarian (*n* = 38)	15 (39.5)	1	1.1–6.3	0.02
	Non-vet (para professional/quack/ farmer himself) (*n* = 52)	33 (63.5)	2.7		
Floor condition	Dry (*n* = 78)	37 (47.4)	1		
	Wet (*n* = 12)	11 (91.7)	12.2	1.5–90.0	0.004
Sunlight accessibility in the cattle shed	Yes (*n* = 86)	45 (52.3)	1		
	No (*n* = 4)	3 (75)	2.7	0.3–27.3	0.37

**Table 2:** Prevalence of *Campylobacter* spp. in dairy cattle herds/farms and different types of samples in two districts (Dhaka and Mymensingh).

Variable	Positive	Prevalence (%)	95% Confidence Interval	*p* Value
Number of herd/farms (*N* = 90)	48	53.3	42.5–63.9	-
**District**				
Mymensingh (*n* = 50)	28	56	41.3–70	0.57
Dhaka (*n* = 40)	20	50	33.8–66.2	
**Sub-districts/city corporation area**				
Sadar Mymensingh (*n* = 26)	15	57.7	36.9–76.6	0.64
Muktagacha (*n* = 6)	2	33.3	4.3–77.7	
Trisal (*n* = 6)	5	83.3	35.9–99.6	
Bhaluka (*n* = 4)	2	50.0	6.8–93.2	
Gouripur (*n* = 3)	2	66.7	9.4–99.2	
Fulbaria (*n* = 5)	2	40.0	5.3–85.3	
Savar (*n* = 14)	7	50.0	23–77	
Dhamrai (*n* = 2)	2	100.0	15.8–100	
Dhaka City Corporation (*n* = 24)	11	45.8	25.5–67.1	
**Sample type**				
Feces (*n* = 540)	167	30.9	27–35	0.000
Milk (*n* = 180)	3	1.7	0.3–4.8	
Feed (*n* = 90)	0	0	0–4	
Water (*n* = 90)	0	0	0–4	
Manure swab (*n* = 90)	14	15.6	8.8–24.7	
Hand-rinse water of animal attendants (*n* = 90)	10	11.11	5.5–19.5	
Overall (*N* = 1080)	194	18	15.7–20.4	
**Animal category**				
Calves (*n* = 180)	51	28.3	21.9–35.5	0.0008
Heifers (*n* = 180)	42	23.3	17.4–30.2	
Cows (*n* = 180)	74	41.1	33.8–48.7	
Total sample (*N* = 540)	167	30.9	27–35	
**Season**				
Pre-monsoon (March–May) (*n* = 300)	87	29	23.9–34.5	0.47
Monsoon (June–October) (*n* = 156)	54	34.6	27.2–42.6	
Winter (November–February) (*n* = 84)	26	31	21.3–42	

*n* = number of herds, samples (in each category), *N* = total number of farms/samples, CI = confidence interval.

**Table 3:** Univariable analysis of biosecurity-related factors of the herd-level *Campylobacter* spp. positivity status in farmed cattle farms of Dhaka and Mymensingh districts.

Variables	Category	Number of Positive Farms (%)	OR	95% CI	*p* -Value
Cleaning and disinfection practices (floor cleaning, cleaning of manger, and drink regularly)	Good practices (*n* = 60)	26 (43.3)	1		
	Poor/no practices (*n* = 30)	22 (73.3)	11.2	3.5–36.4	0
Worker boot disinfection	Yes (*n* = 12)	6 (50)	1		
	No (*n* = 78)	42 (53.8)	1.2	0.3–3.9	0.8
Isolation of animal	Yes (*n* = 19)	9 (47.4)	1		
	No (*n* = 71)	39 (55)	1.3	0.5–3.7	0.55
Access of other animals (poultry/goats/sheep/wild animals) in the farm	No (*n* = 58)	26 (44.8)	1		
	Yes (*n* = 32)	22 (68.7)	2.7	1.1–6.7	0.03
Udder cleaning	With antiseptic (*n* = 14)	8 (57.1)	1		
	With water (*n* = 76)	40 (52.6)	0.8	0.2–2.6	0.75
Manure storage	Solid (*n* = 32)	15 (46.9)	1		
	Semi-solid(*n* = 58)	33 (56.9)	1.5	0.6–3.6	0.36
Animal roams outside of the farm	No (*n* = 21)	1 (4.7)	1		
	Yes (*n* = 69)	47 (68.1)	42.7	5.4–339.0	0
Cattle feces use purpose	Fertilizer (*n* = 41)	22 (53.7)	1		
	Aquaculture (*n* = 49)	26 (53.1)	0.9	0.4–2.2	0.95
History of diarrhea in the farmed cattle	No (*n* = 84)	45 (53.6)	1		
	Yes (*n* = 6)	3 (50)	0.9	0.1–4.5	0.86
Interface (Share same premices with cattle)	No (*n* = 70)	40 (57.1)	1		
	Yes (*n* = 20)	8 (40)	0.5	0.2–1.4	0.175

OR: odds ratio, CI: confidence interval.

**Table 4:** Multivariable logistic regression analysis of potential risk factors with the herd-level *Campylobacter* spp. positivity status in cattle farms of Dhaka and Mymensingh districts.

Risk Factors	Category	AOR	95% CI	SE	*p* Value
Age of the farm	1–5 years	1			
	>5 years	10.6	1.9–59.8	0.882	0.0007
Animal shed	Newly constructed	1			
	Old	4.0	0.8–19.9	0.82	0.09
Training	Yes	1			
	No	3.9	0.7–21.2	0.861	0.112
Knowledge	Yes	1			
	No	3.5	0.4–28.5	1.06	0.23
Cleaning and disinfection practices	Good practices	1			
	No/minimum practices	12.4	2.1–71.6	0.893	0.0048
Floor Condition	Dry	1			
	Wet	2.0	0.1–56.3	1.69	0.67
Animals roaming outside	No	1			
	Yes	44.0	3.6–537.10	1.27	0.003
Other animal (poultry/goat/sheep/wild animal) access	No	1			
	Yes	3.1	0.6–16.1	0.84	0.178
Animal health service provider	Registered veterinarian	1			
	Quack/farmer himself	3.1	0.6–16.3	0.84	0.174

AOR: adjusted odds ratio; CI: confidence interval; SE: standard error.

**Table 5:** Primers and conditions used for the various PCR assays and sequences.

Primers	Sequence (5′-3′)	Target/Purpose	Amplicon Size (bp)	PCR Condition (30 cycle)			Reference
				Denaturation	Annealing	Extension	
16S9F 16S1540R	GAGTTTGATCCTGGCTC AAGGAGGTGATCCAGCC	16S rRNA	1530	94 °C, 30 s	47 °C, 30 s	72 °C, 90 s	[18]
HIP400F HIP1134R	GAAGAGGGTTTGGGTGGTG AGCTAGCTTCGCATAATAACTTG	*hipO* gene	735	94 °C, 30 s	55 °C, 30 s	72 °C, 45 s	[77]
Cj-CdtAU2 Cj-CdtAR2	AGGACTTGAACCTACTTTTC AGGTGGAGTAGTTAAAAACC	*CjcdtA*	631	94 °C, 30 s	53 °C, 30 s	72 °C, 30 s	[78]
Cc-CdtAU1 Cc-CdtAR1	ATTGCCAAGGCTAAAATCTC GATAAAGTCTCCAAAACTGC	*CccdtA*	329				
Cf-CdtAU1 Cf-CdtAR1	AACGACAAATGTAAGCACTC TATTTATGCAAGTCGTGCGA	*CfcdtA*	489				
16S520F 16S1199F 16S741R 16S1240R	GTGCCAGCAGCCGCGG GCAACGAGCGCAACCC GTATCTAATCCTGTTTGC CCATTGTAGCACGTGT	Sequence for Cj-, Cc- and Cf- 16S rRNA	NA	NA	NA	NA	[16]

Cj, *C. jejuni*; Cc, *C. coli*; Cf, *C. fetus*; NA, not applicable.

## Data Availability

The data presented in this study are contained in this manuscript and supplementary materials.

## Supplementary Materials

The following are available online at https://www.mdpi.com/2076-0817/10/3/313/s1, Figure S1: Molecular identification including (a) detection of *Campylobacter* spp. by 16S rRNA gene; (b) confirmation of *C. jejuni* by hippuricase (*hipO*) gene-based PCR, lanes 1 and 17: 100 bp DNA ladder (Promega, USA), lanes 2–14: representative positive isolates, lane 15: positive control (*C. jejuni* ATCC 33560), lane 16: negative control (*Escherichia coli* ATCC 25922); and (c) confirmation of *C. jejuni*, *C. coli*, and *C. fetus* by *cdtA* gene-based multiplex PCR assay, lanes 1 and 17: 100 bp DNA ladder (Promega, USA), lanes 2–12: representative positive isolates, lane 13: positive control (*C. coli* ATCC 33559), lane 14: positive control (*C. jejuni* ATCC33560), lane 15: positive control (*C. fetus* ATCC 27374), and lane 16: negative control (*Escherichia coli* ATCC25922). Figure S2: Schematic diagram of sample collection and testing workflow. Table S1: District and sub-district-wise sample collection status and test results. Table S2: Herd-level data. Table S3: Questionnaire on assessment of herd-level risk factors in dairy farms for *Campylobacter* infection in selected districts of Bangladesh.
