# Supplementary material for: Prevalence, Risk Factors, and Molecular Detection of Campylobacter in Farmed Cattle of Selected Districts in Bangladesh
Source: Pathogens. 2021 Mar 7;10(3):313. doi: 10.3390/pathogens10030313 (PMC7998914; doi:10.3390/pathogens10030313)
Supplement: Supplementary file 1 [file pathogens-10-00313-s001.zip › pathogens-1092805-supplementary materials/Supplementary Table S1 appendix.docx]

**Supplementary Table S1 appendix: District and sub-district-wise sample collection status and test results**

| **District** | **Sub-district/**  **City corporation** | **Month of sampling** | **Animal sample** | | **Environmental and other samples** | | | |  |
| --- | --- | --- | --- | --- | --- | --- | --- | --- | --- |
|  |  |  | **No of feces sample**  **(positive)** | **No of milk sample**  **(positive)** | **No of feed sample**  **(positive)** | **No of water (positive)** | **No of manure (positive)** | **No of hand rinse water (positive)** | **Total (positive)** |
| Mymensingh | Sadar | Apri-May 2018 | 156(46) | 52(2) | 26(0) | 26(0) | 26(3) | 26(2) | 312(53) |
|  | Muktagacha | Jun-Aug 2018 | 36(11) | 12(0) | 6(0) | 6(0) | 6(2) | 6(2) | 72(15) |
|  | Trisal | Jun-Aug 2018 | 36(20) | 12(0) | 6(0) | 6(0) | 6(0) | 6(1) | 72(21) |
|  | Bhaluka | Dec 2018–Feb 2019 | 24(9) | 8(0) | 4(0) | 4(0) | 4(1) | 4(0) | 48(10) |
|  | Gouripur | Dec 2018–Feb 2019 | 18(5) | 6(0) | 3(0) | 3(0) | 3(1) | 3(0) | 36(6) |
|  | Fulbaria | Dec 2018–Feb 2019 | 30(9) | 10(0) | 5(0) | 5(0) | 5(1) | 5(1) | 60(11) |
|  | Sub-total |  | 300(100) | 100(2) | 50(0) | 50(0) | 50(8) | 50(6) | 600(116) |
| Dhaka | Savar | Jun-August 2019 | 84(23) | 28(0) | 14(0) | 14(0) | 14(2) | 14(2) | 168(27) |
|  | Dhamrai | December 2019-February 2020 | 12(3) | 4(0) | 2(0) | 2(0) | 2(0) | 2(0) | 24(3) |
|  | Dhaka city corporation | March-May 2020 | 144(41) | 48(1) | 24(0) | 24(0) | 24(4) | 24(2) | 288(48) |
|  | Subtotal |  | 240(67) | 80(1) | 40(0) | 40(0) | 40(6) | 40(4) | 480(78) |
|  | Total |  | 540(167) | 180(3) | 90(0) | 90(0) | 90(14) | 90(10) | 1080(194) |
