# Supplementary material for: Prevalence, Risk Factors, and Molecular Detection of Campylobacter in Farmed Cattle of Selected Districts in Bangladesh
Source: Pathogens. 2021 Mar 7;10(3):313. doi: 10.3390/pathogens10030313 (PMC7998914; doi:10.3390/pathogens10030313)
Supplement: Supplementary file 1 [file pathogens-10-00313-s001.zip › pathogens-1092805-supplementary materials/Supplementary Table S3 appendix.docx]

**Supplementary Table S3 appendix: Questionnaire on assessment of herd level risk factors in dairy farms for Campylobacter infection in selected districts of Bangladesh**

| **Questionnaire ID:………** | |  |  |  | |  |  |  | |  |  | |
| --- | --- | --- | --- | --- | --- | --- | --- | --- | --- | --- | --- | --- |
| **Date of interview: …./…../20…….** | |  |  |  | |  |  |  | |  |  | |
| **Section A: Farm environment and management factors** | | | | | | | | | | |  |  |
| **1. Location of the farms** | | |  |  | |  |  |  | |  |  | |
| Village:……… | | Upazila:…….. | |  | | District: | |  | |  |  | |
|  |  |  |  |  | | - Dhaka[0] | - Mymensingh[1] |  | |  |  | |
| **2. Age of the farms** | |  |  |  | |  |  |  | |  |  | |
|  | - 1-5 years [1] | - >5 years [0] | | | |  |  |  | |  |  | |
| **3. Animal shed** | |  |  |  | | **4. Size of the farm** | |  | |  |  | |
|  |  |  |  |  | |  |  |  | |  |  | |
| - Newly build(within a year) [1] | | |  |  | | - Up to 20 cattle [1] | |  | |  |  | |
| - Old (more than one year) [0] | | |  |  | | - More than 20 cattle[0] | |  | |  |  | |
|  |  |  |  |  | |  |  |  | |  |  | |
| **5. Stocking density of the farm** | | | |  | | **6. Milking procedure** | | |  | |  | |
|  |  |  |  |  | |  |  |  | |  |  | |
| - up to 50 square feet/cow[0] | | |  |  | |  | - Hand milking [0] | | | | | |
| - More than 50 square feet/cow[1] | | |  |  | |  | - Machine milking [1] | | | | | |
|  | |  |  |  | |  |  |  | |  |  | |
| **7. Type of feed use** | | |  |  | | **8. Training of farmers/animal attendants on cattle husbandly and best farm practices** | | | | |  |  |
| - Ready-made commercial feed [1] | | |  |  | |  | - Yes [1] |  | |  |  | |
| - Prepared by farmer [0] | |  |  |  | |  | - No[0] |  | |  |  | |
| **9. Knowledge on risk perception to cattle get infection if freely roams** | | | | | **10. Cattle handler type** | | | |  | |  | |
|  |  |  |  |  | |  |  |  | |  |  | |
| - Yes[1] |  |  |  |  | | - Family member [1] | |  | |  |  | |
| - No[0] |  |  |  |  | | - Employee [0] | |  | |  |  | |
|  |  |  |  |  | |  |  |  | |  |  | |
| **11. Prophylactic use of antibiotic in feed** | | | |  | | **12. Veterinary health care facilities ensured by** | | | | |  |  |
|  |  |  |  |  | |  |  |  | |  |  | |
| - Yes [1] |  |  |  |  | | - Registered veterinarian [1] | | |  | |  | |
| - No[0] |  |  |  |  | | - Quack/paravet/farmers[0] | | |  | |  | |
|  |  |  |  |  | |  |  |  | |  |  | |
| **13. Floor condition** | |  |  |  | | **14. Sun light accessibility in the farm** | | |  | |  | |
| - Dry [1] | |  |  |  | | - Yes [1] | |  | |  |  | |
| - Wet [0] | |  |  |  | | - No[0] | |  | |  |  | |
|  |  |  |  |  | |  |  |  | |  |  | |

| **Section B: Biosecurity and cleaning disinfection related practices** | | | | | | | | | | | | | | |
| --- | --- | --- | --- | --- | --- | --- | --- | --- | --- | --- | --- | --- | --- | --- |
| **1. Cleaning and disinfection practices (floor cleaning, cleaning of manger and drinker regularly)** | | | | | | | | | | | | |  | |
|  |  |  | |  | |  |  | | |  | |  | |  |
| - - Good practices[1] | | |  | | |  |  | |  | |  | |  | |
| - - Poor/no practices [0] | | |  | | |  |  | |  | |  | |  | |
|  |  |  | |  | |  |  | | |  | |  | |  |
| **2. Workers boot disinfection** | | |  | | | **3. Isolation/quarantine facilities if animal got sick /new arrival** | | | | | | | | |
|  | - Yes [1] |  | |  | |  | - Yes [1] | | |  | |  | |  |
|  | - No[0] |  | |  | |  | - No[0] | | |  | |  | |  |
|  |  |  | |  | |  |  | | |  | |  | |  |
| **4.Other animal (poultry/goat/sheep/wild animal) access** | | | | | | | **5. Udder cleaning** | | | |  | |  | |
|  |  |  | |  | |  |  | | |  | |  | |  |
|  | - Yes [0] |  | |  | |  | - Yes [1] | | |  | |  | |  |
|  | - No[1] |  | |  | |  | - No[0] | | |  | |  | |  |
| **6. Manure storage** | |  | |  | |  | **7. Animal roams outside of the farm** | | | | | | | |
|  | - Solid [1] |  | |  | |  | - Yes [0] | | |  | |  | |  |
|  | - Semi-solid [0] | |  | | |  | - No[1] | |  | |  | |  | |
|  |  |  | |  | |  |  | | |  | |  | |  |
| **8.Cattle faces use purpose** | | |  | | |  | **9. History of diarrhea in the herd** | | | | | | | |
|  |  |  | |  | |  |  | | |  | |  | |  |
|  | - Fertilizer [1] |  | |  | |  | - Yes [0] | | |  | |  | |  |
|  | - Aquaculture[0] | |  | | |  | - No[1] | |  | |  | |  | |
| **10. Interface** | |  | |  | |  |  | | |  | |  | |  |
|  | - Yes [0] |  | |  | |  |  | | |  | |  | |  |
|  | - No[1] |  | |  | |  |  | | |  | |  | |  |
| **Section C: Infection status of the farm (feces examination by laboratory tests)** | | | | | | | | | | | | | | |
|  | - Yes [0] | - Number of animal……….. | | | | | | | | | | | | |
|  | - No [1] | - Cow… | | | - Heifer… | | | - Calf… | | | | | | |
