# Supplementary figures and images for: Prevalence, Risk Factors, and Molecular Detection of Campylobacter in Farmed Cattle of Selected Districts in Bangladesh
Source: Pathogens. 2021 Mar 7;10(3):313. doi: 10.3390/pathogens10030313 (PMC7998914; doi:10.3390/pathogens10030313)

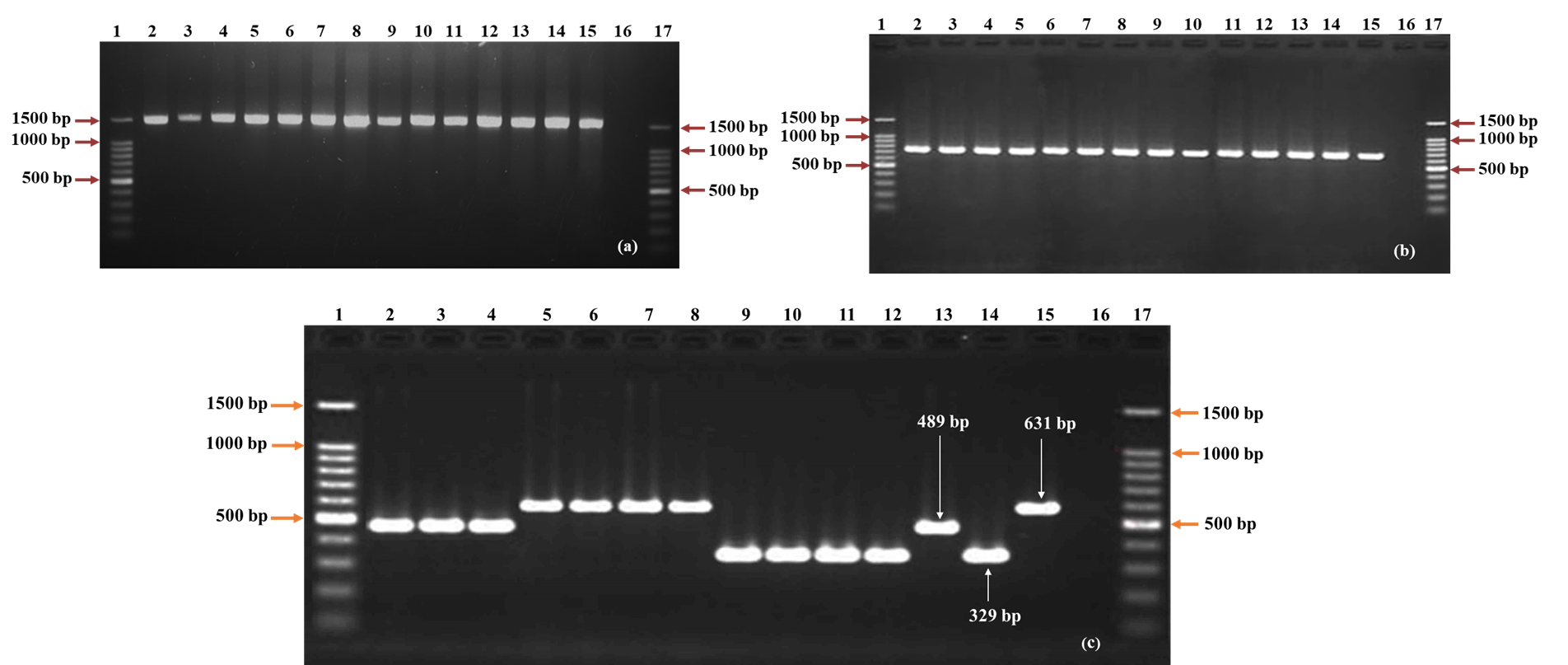

Supplement: Supplementary file 1 [file pathogens-10-00313-s001.zip › pathogens-1092805-supplementary materials/Supplementary Figure S1.tif]

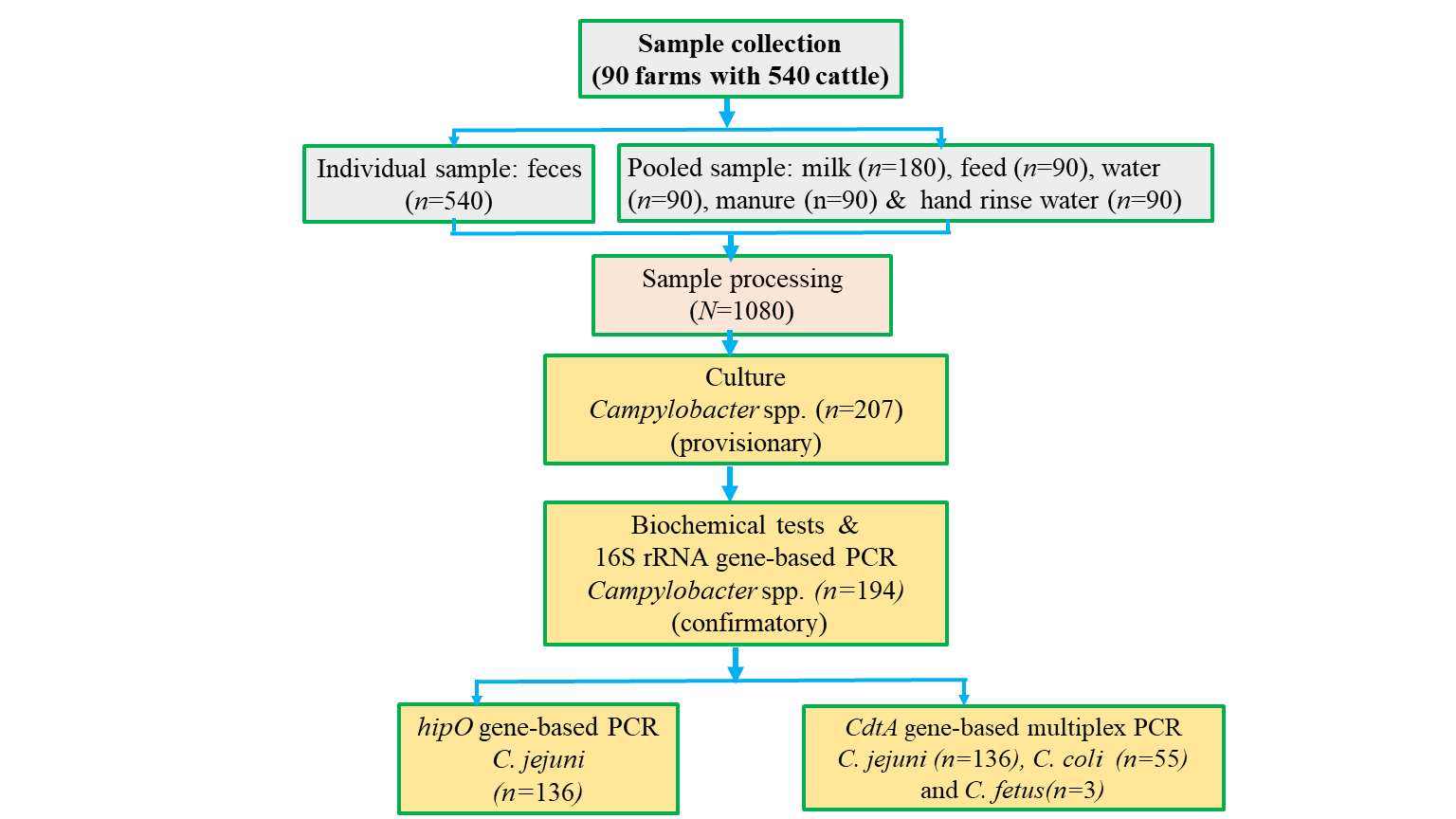

Supplement: Supplementary file 1 [file pathogens-10-00313-s001.zip › pathogens-1092805-supplementary materials/Supplementary Figure S2.tif]
